# PrEP reminds me that I am the one to take responsibility of my life*:* a qualitative study exploring experiences of and attitudes towards pre-exposure prophylaxis use by women in Eswatini

**DOI:** 10.1186/s12889-021-10766-0

**Published:** 2021-04-14

**Authors:** Pia Juul Bjertrup, Nqobile Mmema, Velibanti Dlamini, Iza Ciglenecki, Qhubekani Mpala, Sindy Matse, Bernhard Kerschberger, Alison Wringe

**Affiliations:** 1 Médecins Sans Frontières, Mbabane, Eswatini; 2 Department of Anthropology, University of Copenhagen, Øster Farimagsgade 5, 1353 Copenhagen, Denmark; 3 Médecins Sans Frontières, Geneva, Switzerland; 4 Eswatini National AIDS Programme, Ministry of Health, Mbabane, Eswatini; 5 Department of Population Health, London School of Hygiene and Tropical Medicine, London, UK

**Keywords:** Eswatini, Qualitative research, Pre-exposure prophylaxis, HIV, Self care, Women

## Abstract

**Background:**

Pre-exposure-prophylaxis (PrEP) has been heralded for its potential to put women in control of preventing HIV infection, but uptake and continuation rates have been disappointing in high-incidence settings in sub-Saharan Africa. We explored structural and social factors that influenced PrEP use among young women and pregnant or breastfeeding women in rural Eswatini.

**Methods:**

We conducted two in-depth interviews with ten women on PrEP, and one-time in-depth interviews with fourteen women who declined or discontinued PrEP. Interviews covered decision-making processes around PrEP initiation and experiences with pill-taking. In-depth interviews were conducted with nine health workers, covering experiences in delivering PrEP services, and two focus group discussions were held with men to elicit their perceptions of PrEP. Interviews and discussions were audio-recorded, translated, transcribed and analysed thematically, using an inductive approach.

**Results:**

PrEP initiation and use were experienced by many women as empowering them to take control of their health and well-being, and stay HIV free, facilitating them to realise their aspirations relating to motherhood and educational attainment. However, the social norms that defined relationship dynamics with partners or family members either undermined or promoted this empowerment potential. In particular, young women were rarely supported by family members to take PrEP unless it was perceived to be for protecting an unborn child. Stigmatisation of pill-taking through its associations with HIV and the burden of daily pill-taking also contributed to PrEP discontinuation.

**Conclusions:**

Unlike many prevention tools, PrEP enabled women to achieve a sense of control over their lives. Nevertheless, women’s agency to continue and adhere to PrEP was influenced by social and structural factors including gender norms, family expectations of young women, relationship dynamics and stigma related to HIV. Future interventions should address these barriers to promote PrEP use among sexually-active women.

## Background

The HIV epidemic in Eswatini (formerly Swaziland) remains a public health concern, with persistently high levels of incidence, particularly among women of reproductive age [1–3]. By 2018, HIV prevalence was 27% in the general adult population compared with 35% among women, and annual HIV incidence was 1.2 per 100 person-years among women aged 15–49 years, compared to 0.8 per 100 person-years in men [1]. HIV estimated incidence is also higher in young women aged 15–24 years at 1.9 per 100 person-years, compared to their male peers among whom it reaches 0.8 per 100 person-years [3], demonstrating the need for HIV preventive measures for adolescent girls and young women (AGYW) in this setting. Furthermore, biological changes during pregnancy and the postpartum period, such as hormonal and immune responses, increase HIV susceptibility among women [4, 5]. This increased susceptibility together with the risk of mother-to-child transmission makes pregnant and breastfeeding women (PBW) an important target groups for HIV preventive measures including pre-exposure prophylaxis (PrEP).

The widespread provision of antiretroviral therapy (ART) and behavioural interventions such as promoting partner reduction and condom use have been insufficient for HIV epidemic control, even when coupled with biomedical prevention interventions such as male circumcision [6–9]. In 2015, the World Health Organization (WHO) recommended PrEP for people at high risk of HIV acquisition, defined as groups with incidence above 3 per 100 person-years [10], following several studies demonstrating its effectiveness in reducing HIV infections among high-risk populations [11, 12].

Female-initiated biomedical HIV prevention technologies such as PrEP have been heralded as offering new possibilities for women to reduce their HIV risks by giving them control over the decision to use them [13]. However, the promotion of ART prevention approaches (e.g. PrEP, prevention of mother-to-child transmission) also imbues the person taking the medication with a responsibility to care for their own and others’ health that can be either empowering or a burden [14–17]. Despite the empowering and agential potential of health technologies, studies show how beneficiaries struggle to take on the necessary responsibilities, potentially leading to an exclusion of those unable to become sufficiently resilient [15, 17, 18]. In this vein, Rose and Lentzos (2017) argue for a greater understanding of the collective conditions, power and resources that are necessary to ensure that responsibility and resilience become a reality [19]. It is only when these conditions are met that PrEP can move beyond being simply a medical prescription, towards achieving its potential as a tool to enable women to enact the agency that is necessary for securing their health and quality of life on a daily basis [16].

In practice, social barriers including HIV stigma and structural factors such as those that impede regular access to health facilities [18, 20–23] may undermine the empowerment potential of PrEP, but few studies have explored how women experience these opposing dynamics. In this context, we explored the structural and social factors that influence experience of PrEP use and continuation among AGYW and PBW.

## Methods

### Study setting

Since 2007, Médecins sans Frontières (MSF), in collaboration with the Ministry of Health, has been providing HIV care in the predominantly rural Shiselweni region of Eswatini. In 2017, a PrEP demonstration project investigated PrEP provision among the general population. A nested qualitative study explored the structural and social factors that influenced PrEP experiences of AGYW aged 16–25 years and PBW aged ≥16 years to inform national PrEP roll-out.

### Sampling and recruitment

We purposively sampled participants for IDIs to include clients with a variety of PrEP histories. Sampling included AGYW or PBW who i) had been on PrEP for up to 6 months; ii) had initiated PrEP in the previous month; iii) had been offered PrEP but declined to initiate; iv) discontinued PrEP at the first monthly visit at the health facility; v) discontinued PrEP after the first prescription without returning to the health facility (Table 1).

**Table 1:** Respondent characteristics of PrEP clients (*n* = 24 respondents)

Age, years	N (%)
18–20	7 (29)
21–25	12 (50)
26–30	3 (13)
31–35	2 (8)
PrEP status	
Declined PrEP	5 (21)
On PrEP < 1 month	9 (38)
On PrEP > 1 month	1 (4)
Discontinued PrEP	9 (38)
Relationship status	
Multiple partners	3 (13)
Partner, living together	3 (13)
Partner, not living together	17 (71)
Single	1 (4)
Status of partner(s)	
Known HIV-serodiscordant relationship	6 (25)
Non−/unknown HIV-serodiscordant relationship	18 (75)
Children	
No children	2 (8)
Pregnant	3 (13)
1 child	11 (46)
2 or more children	8 (34)
Educational level	
Primary	6 (25)
Secondary	14 (58)
Tertiary	2 (8)
Not specified	2 (8)
Employment status	
Employed	1 (4)
Unemployed	16 (67)
In school	7 (29)

HIV counsellors recruited AGYW and PBW, explaining the study before seeking permission for the research team to phone them to schedule an interview. Men for FGDs were recruited during health promotion sessions targeting men. Health workers involved in the delivery of PrEP services were purposively sampled from four facilities, ensuring the inclusion of different cadres and roles (Table 2).

**Table 2:** Respondent characteristics of healthcare workers (*n* = 11)

Gender	N (%)
Female	7 (64)
Male	4 (36)
Job title	
Nurse	4 (36)
HIV testing lay counsellors	6 (55)
Community mobiliser	1 (9)
Time in job role, years	
2–4	3 (27)
5–7	5 (45)
8–10	1 (9)
> 10	2 (18)

### Data generation

We conducted two rounds of in-depth interviews (IDIs) with seven PBW and three AGYW who were not pregnant or breastfeeding. The first interview covered their personal circumstances, reasons for initiating PrEP and early experiences with pill-taking, while the second interview covered later experiences with PrEP and any evolution in their personal circumstances. We conducted one-time IDIs with 14 AGYW and PBW who declined or discontinued PrEP, exploring reasons for these decisions, and with 11 healthcare workers to explore their experiences of providing PrEP services and their suggestions for improving PrEP uptake and retention. Interviews lasted approximately 60–90 min, and were conducted in a private place of the participant’s choice by the lead author, a trained anthropologist (PB), and/or a local qualitative research assistant (NM or VD), matched to the sex of the participant. After each interview, notes and observations from interviews were written up and debriefing sessions were held between the interviewing team.

We conducted two focus group discussions (FGDs) with 11 men from the community, exploring perceptions of and attitudes towards PrEP. These were held in the community, facilitated by the two local research assistants (VD and NM) and lasted approximately 1 h. The age range of the men was 18 to 60 years of age.

### Data analysis

IDIs and FGDs were conducted in SiSwati and audio-recorded following informed written consent. Audio-recordings were translated and transcribed into English.. Thematic analysis was conducted whereby transcripts, fieldworker notes and observation templates were coded iteratively and inductively in NVivo10 by PB, NM and VD. Codes were progressively incorporated into a coding framework that was developed continually to enable inclusion of emerging categories, and analytical memos were written to aid the process of raising findings to a conceptual level and to explore relationships between emerging themes.

## Results

### HIV risks

Most women felt at risk of HIV infection due to relationships that they characterized as lacking mutual trust, unreliable and unstable, as well as difficulties negotiating condom use with their partners. Women described how sexual relationships and encounters were difficult to predict and control – for example, due to having a migrant partner who would turn up unannounced or because they were linked to economic reasons. Unplanned, and often unwanted, pregnancies made women feel dependent on their child’s father as a breadwinner, particularly as most women were unemployed and unable to provide for a child alone. This economic dependence inclined women to stay in relationships where they felt at risk of HIV.

### PrEP as an enactment of agency and self-care

Women highlighted several ways through which PrEP represented an enactment of agency, enabling them to take control of HIV risks and protect themselves and/or their baby from HIV infection. It afforded them peace of mind and confidence that they would not become infected, which in turn would ensure that they could stay healthy and see their children grow up. This motivation could lead them to overcome financial barriers to clinic attendance:

*I’m committed to this [PrEP]. Even if I don’t have transport money, I’ll rather try borrowing it from neighbours, so I’m able to go to the clinic to get my PrEP pills. Then I can raise my children properly and not end up having HIV (Woman 21–25 years).*

Continually testing HIV-negative, when coming to follow-up visits at the clinic, reaffirmed women’s confidence in their choice to initiate PrEP. Most women described the decision to initiate PrEP as being their own to take, appreciating the autonomy that this afforded them by removing the need to negotiate with their partners as was the case with condoms. However, some were later confronted by partners who felt they should have been included in the decision.

PrEP use also evoked a sense of responsibility and care for one’s own life and self-worth. Some women recounted how PrEP had served as a reminder of the importance of taking care of themselves, and had even encouraged them to negotiate condom use in their relationship again:

*Now when we have sex, we use condoms. Before I started PrEP, we weren’t using condoms. The pill encourages me to use condoms and to know that I will not end up getting infected (Woman 18–20 years).*

For AGYW, both PrEP and contraceptive pills were seen as items that would enable them to fulfil future educational and career aspirations, essential for the financial independence that many craved, and that they believed would be undermined if they seroconverted or fell pregnant:

*I want to finish school, while not having a baby and I did PrEP because I don’t want to be HIV-positive. […*] *I do not trust love; rather I trust that PrEP and Jadelle [contraceptive implant] will help me (Woman 18–20 years).*

### “PrEP for life” and pill fatigue

While PrEP enabled some to exert a degree of agency in their sexual lives, the unpredictability and lack of control that characterized the women’s sexual relationships led them to perceive their need for PrEP as lifelong or continuous until they found a stable partner or got married. This in turn could lead to pill fatigue and discontinued use, with some PrEP using participants starting to question the relative advantages of taking antiretroviral (ARV) drugs for prevention over taking ARVs for treatment, given the perceived similarity in the pill-taking burden:

*I was tired of the pills, they are big and like ARVs. You have to take them every day and they are difficult to swallow. I felt like I have to take it for the rest of my life […] It is like I already have the virus (Woman 21–25 years).*

All the women in HIV-serodiscordant relationships stated that their partner was on ART. However, some did not seem to know that this would reduce their HIV infection risk, nor did they know their partner’s viral load (VL) results. Furthermore, a few of the women who were interviewed expressed uncertainty as to whether their partners were taking ARVs as prescribed, and understood that this perceived lack of responsibility on the part of their partner increased the risk of acquiring HIV:

*I feel at risk, even though he is taking the pills [ARVs], I am not convinced I am safe. He is not responsible. Sometimes he goes somewhere and comes home late and then he will not be able to take his pills (Woman 21–25 years).*

Healthcare workers suggested that they started clients on PrEP if they were in HIV-serodiscordant relationships without further inquiry as to the HIV-infected partner’s VL result. They sometimes struggled to assess clients’ current risks when providing guidance on PrEP duration for their clients, opting for a perceived simpler and clearer message of the need for PrEP “for life”:

*I think PrEP is for everyone, for life, because we are exposed every day. If we were to say you have been taking PrEP for 2 years you think you’re safe now. Then for 5 months you don’t take it, then you expose yourself [to risk] (Health care worker).*

### Social relations and their interactions with women’s agency to use PrEP

Although many women explained that decisions to take PrEP could afford them with a sense of autonomy over their lives, a reported lack of shared decision-making, or a perceived inability to disclose the decision to a partner or family members could lead to challenges in continuing PrEP. Women reported hiding their drugs under mattresses or other places where nobody would find them. This lack of visibility made some women forget to take their pills, or made it difficult to do so in private:

*It would be easier to take the pills if they knew about it at home. I need to do everything in secrecy. If they knew, there would maybe be someone to remind me of the pills […] I have hidden my pills and I need to look over my shoulder before taking it. If they knew, I would keep the pills wherever I wanted, so when I get to my bedroom, I see my pills and that would remind me to take them (Woman 18–20 years).*

Women who had not disclosed PrEP use to sexual partners stated that taking PrEP was challenging when they spent the night at their partner’s place, as they feared being caught with the tablets. They feared negative responses from partners, such as being perceived as promiscuous or HIV-positive. Around half of the participants reported that they had disclosed PrEP use to a sexual partner and some stated how this led to arguments:

*We quarrelled because of PrEP. It is as if he hears from his friends that PrEP is for prostitutes. What is the need for me to take PrEP? (Woman 18–20 years).*

During focus group discussions, some male participants suggested that PrEP use by their partners might provoke feelings of insecurity and lack of trust within a relationship and that they may interpret its use as an accusation that they were infected with HIV, which could in turn incline them towards physical violence against their partners:

*She must be thinking I have AIDS, that could annoy me and I could end up beating her. (Male participant 1, FGD)...*

*He is right because I might be led to think that when she is protecting herself, it is not because she has many sexual partners. […] It could bring insecurity. (Male participant 2, FGD).*

On the other hand, all women in HIV-serodiscordant relationships stated that their partners supported them being on PrEP, which was also reported during FGDs with men where it was seen to be appropriate:

*“If I know I’m HIV-positive then I’ll understand that this person is actually doing it for her safety and my safety. By taking PrEP, she’s actually thinking for both of us. We will both benefit” (FGD with men).*

Disclosing PrEP use to co-habiting family members could lead to encouragement and pill reminders, which promoted adherence, especially among those who had children or were pregnant. In many cases, women were celebrated for taking responsibility and care for themselves and their child (ren). For some women, however, disclosing PrEP use to co-habiting family members meant being on the receiving end of negative comments and attitudes that could undermine their motivation to continue PrEP. Young women without children found it particularly difficult to disclose PrEP use to parents or other parental authorities at home, as they feared being sexually active as a young, unmarried women would be judged morally unacceptable:

*My father and my mother, I did not tell them […] I don’t know if they know I’m sexually active. My parents can really give me problems. They can say: ‘as young as you are, you are taking these pills, and how do you live your life?!’ (Woman 21–25 years).*

Parental resistance towards PrEP was also mentioned by healthcare workers, who were sometimes confronted with angry parents during morning talks on PrEP in the clinic. According to health workers, parents were reportedly opposed PrEP as they perceived that it could encourage teenage children to be sexually active, rather than protecting them from HIV infection risk through abstinence:

*During health talks, there is the challenge that some parents aren’t able to accept. They say that if they find these pills in the bedroom of the child, they might chase the child away from home. ‘It is in our nature as parents, we cannot accept that our child is having sex’ and yet the child is just protecting herself (Health care worker).*

Healthcare workers also mentioned how reaching young people with PrEP was difficult, as they would rarely come to the healthcare clinic, except young pregnant women who came for antenatal care.

## Discussion

This study showed how an array of structural and social factors influenced women’s agency to effectively harness the prevention potential offered by PrEP. While PrEP enabled women to achieve a sense of autonomy over their own or their baby’s risk of acquiring HIV and encouraged a sense of self-worth and possibilities for self-care, its longer-term use was often undermined by pill fatigue, family expectations of young women, gender norms, relationship dynamics and stigma related to HIV, over which the women had less influence.

While PrEP was perceived to afford some autonomy over HIV risks, as found elsewhere [18], our results also highlight challenges in assuming responsibility for daily pill taking “alone”, without support from family members or partners, as was often the case for young women. In alignment with other studies from the region, we found that when disclosure was pursued, it did not always lead to acceptance or support from the women’s family members or partners, which could trigger PrEP discontinuation [23, 24].

Future access to two-three-monthly injectable PrEP might reduce the need for women to disclose PrEP status, as well as reducing the need for pill-taking reminders by family and partners. A recent study among youth in South Africa demonstrated preference for long-lasting PrEP in injectable or implant forms, highlighting the benefits of the “invisibility” of such products [25, 26], although other studies have demonstrated cases of intimate partner violence following the discovery of monthly rings [27]. Furthermore, longer-lasting interventions do not address the social and structural challenges that contribute to HIV-related stigma that PrEP users face, nor do they bring about the moral and emotional support for being on PrEP that seem important especially for AGYW. To address this, community-level interventions could be carried out promoting PrEP acceptance among women’s partners and families and addressing the social norms that undermine its optimal use. Adherence and support clubs could be beneficial for AGYW on PrEP, regardless of its format, and their effectiveness for PrEP should be investigated [28]. As young women without children often do not attend the health clinic, other out-of-facility models are needed to ensure they access PrEP in combination with family planning measures.

Perceptions of the need for lifelong PrEP were driven by the nature of many women’s relationships which were often characterised as unstable or polygamous. As found elsewhere, among sero-discordant couples, some women were either unaware of the VL status of their partner or did not trust the regularity of their pill-taking [29, 30]. These findings emphasise the importance of regular counselling for PrEP recipients, with greater focus on HIV transmission risks from partners on ART and support to discontinue and reinitiate PrEP as risks evolve. Couple-counselling that focuses on mutual support for ART and PrEP adherence could help reduce the time which women need to remain on PrEP. Health workers also need support to tailor risk assessments to each woman, rather than assuming that their risk is lifelong.

The study had several limitations. It was conducted in the context of a well-resourced demonstration project, and perceptions of PrEP might change with time as the intervention becomes more widely available or in settings where fewer resources are available. Interviews with health workers and women who discontinued or declined PrEP were conducted once and could not capture how their experiences may have evolved. Finally, insights from male partners and family members of AGYW are lacking due to the ethical risks and challenges of recruiting these groups for IDIs in this setting.

## Conclusions

In conclusion, we found that women’s motivations for PrEP were driven by their perceptions of HIV risks and their desire for self-care and control over their HIV risks. However, these aspirations were often undermined by social factors including gender norms within relationships that drove stigmatising associations with pill-taking, or within families where moralising attitudes limited support for PrEP for young women. Further research should identify interventions to effectively address these social and structural barriers to daily PrEP taking and investigate how best to implement injectable PrEP and out-of-facility PrEP, with integration into family planning interventions for AGYW.

## Data Availability

The datasets used and/or analysed during the current study available from the corresponding author on reasonable request.
